# Phonatory function and characteristics of voice in recovering COVID-19 survivors

**DOI:** 10.1007/s00405-022-07419-2

**Published:** 2022-05-15

**Authors:** Dalia G. Yasien, Eman S. Hassan, Hanan A. Mohamed

**Affiliations:** 1 Phoniatric Unit, ENT Department, Helwan University Hospital, Faculty of Medicine, Helwan University, Cairo, Egypt; 2 Phoniatric Unit, ENT Department, Assiut University Hospital, Faculty of Medicine, Assiut University, Assiut, Egypt

**Keywords:** Phonasthenia, Dysphonia, COVID-19 survivors, MPT, Pneumonia

## Abstract

**Introduction:**

This study aimed to evaluate the phonatory function of recovered COVID-19 survivors. The universal outbreak of COVID-19 led to the occurrence of otolaryngological manifestations that raised concerns about the assessment of the phonatory function in recovering patients.

**Methods:**

This is a prospective, cross-sectional, case-controlled study carried out on 364 laboratory-confirmed non-critical COVID-19 survivors and 100 as healthy controls. The study participants were classified into two groups according to the disease severity. Group1 comprised 212 survivors who recovered from pneumonia and group 2 was made up of 152 survivors of severe pneumonia. All patients were subjected to an auditory perceptual assessment of the voice (APA) and Maximum Phonation Time (MPT) measurements.

**Results:**

Phonasthenic manifestations were significantly more frequent in COVID-19 survivors than in controls (*P* < 0.000) with a higher percentage recorded among severe pneumonia survivors (87.5%) than among pneumonia survivors (60.8%) with a *P* value of < 0.01. Dysphonia and excessively soft loudness were significantly more common among survivors than among controls (*P* < 0.002 and *P* < 0.000, respectively) with no significant difference between the patient groups. The MPT was significantly shorter among survivors than among controls (*P* < 0.000). The mean MPT was 15.97 s in the control group, 10.72 s in the pneumonia group, and 8.88 s in the severe pneumonia group, with the differences between the groups being statistically significant (*P* < 0.000), suggesting a higher impairment of lung volume and phonatory function in severe cases.

**Conclusions:**

Phonasthenia, dysphonia, and decreased MPT could be otolaryngological manifestations of COVID-19. Laryngeal function assessment should be considered in COVID-19 survivors.

## Introduction

Coronavirus disease 2019 (COVID-19) is a contagious illness caused by a novel coronavirus called the severe acute respiratory syndrome coronavirus 2 (SARS-CoV-2, formerly called 2019- nCoV) [1]. On March 11, 2020, the World Health Organization (WHO) declared COVID-19 a global pandemic [2].

In the light of commonly documented lung injuries related to COVID-19 [3, 4], concerns are raised about the evaluation of the pulmonary function of discharged patients. Persistent impairments of the pulmonary function and exercise capacity have been known to last for months or even years in recovered survivors of other types of coronavirus pneumonia [5–7]. Voice production depends on a finely balanced relationship between the forces exerted by the intrinsic muscles of the larynx and the forces exerted by the air as it is exhaled from the lung. This occurs through what is called the aerodynamic-myoelectric theory of phonation, which states that the vibration of the vocal folds is induced by a combination of the aerodynamic, muscular, and elastic forces of the larynx [8].

An abnormal pulmonary function and a reduced pulmonary vital capacity affect voice quality and the maximum phonation time (MPT) [9]. Colton and Casper (1990) suggested that the ability to sustain phonation provides information about the laryngeal function and the pulmonary function at the same time. When the pulmonary function is the problem, there will be a reduction in the amount of air needed to support phonation. On the other hand, in the case of laryngeal-level problems, glottal resistance to airflow may increase or decrease due to hyper-adduction or the inadequate closing of the glottis [10]. A recent report presented pulmonary function abnormalities in COVID-19 patients at the time of hospital discharge [11]. However, to the best of our knowledge, there is no available report on the laryngeal function in recovered COVID-19 patients.

Many parameters can be used in describing the laryngeal function. These include the assessment of voice quality, acoustic measurements, and aerodynamic measurements. The MPT is an easy aerodynamic measure as it is rapid, non-invasive, and requires no special equipment.

In this study, we aimed to evaluate the phonatory function of recovered COVID-19 survivors by assessing the voice quality and MPT.

## Patients and methods

### Patients

Three hundred sixty-four laboratory-confirmed non-critical COVID-19 survivors and 100 healthy controls were recruited in this prospective cross-section case–control study that was carried out from July 2020 to April 2021. According to the WHO interim guidance [12], the disease was classified as mild illness (mild symptoms without any radiographic appearance of pneumonia), pneumonia (the presence of both symptoms and radiographic evidence of pneumonia with no requirement for supplemental oxygen), severe pneumonia (the presence of pneumonia with at least one of the following: respiratory rate > 30 cycles/minute, severe respiratory distress, or SpO_2_ ≤ 93% on room air at rest), and critical cases (e.g., respiratory failure requiring mechanical ventilation, septic shock, other organ failures, or admission to the intensive care unit). Two hundred and twelve survivors who recovered from pneumonia (Group 1) and 152 survivors from severe pneumonia (Group 2) were enrolled in this study while mild and critical cases were excluded. We also excluded people with a previous history of voice disorders, asthma, or any form of respiratory disease.

### Methods

Every patient’s full history was taken during an interview. The auditory perceptual assessment of the voice (APA) was evaluated by three phoniatricians after recording the participant’s speech. Speech samples included reading a standardized text or counting and isolated vowel phonation at a comfortable pitch and loudness as naturally as possible. The voice sample was obtained by recording the patient’s voice with an audio recorder in a quiet environment. The patients were asked to phonate a prolonged /a/ vowel sound with normal intensity and pitch. In addition, they were asked to count from one to ten.

Auditory perceptual assessments of voice samples were performed using the protocol of the modified GRBAS scale [13] that evaluates:

- The overall grade (G) of dysphonia: Normal (0), Mild dysphonia (1), Moderate dysphonia (2), or severe dysphonia (3).
- The character (quality) of dysphonia: Strained, Leaky, Breathy, or Irregular.
- The pitch: Overall increased, Decreased, or Diplophonia.
- The register: Modal, Falsetto, or Fry.
- The loudness: Excessively loud, Excessively soft, or Fluctuating.
- The glottal attack: Normal, Soft, or Hard.

Cronbach’s alpha test was used to measure the scale reliability. The overall grading of dysphonia of the three examiners revealed high inter-rater reliability (Cronbach’s Alpha = 0.89).

The MPT was calculated using an audio recorder and stopwatch. This parameter was measured by asking each patient while sitting upright, to take a deep breath and then to sustain the /a:/ vowel sound for as long as possible at his/her comfortable pitch and loudness without straining. The task was repeated three times and the longest duration of the three attempts is considered as the MPT.

### Ethical considerations

The study was approved by the research ethics committee of the Faculty of Medicine-Helwan University. All participants gave their written informed consent to participate in the study.

### Statistics

The data were entered into Statistical Package for the Social Sciences version 24 and the same software program was used for data analyses. Quantitative variables were presented as mean values with standard deviations for normally distributed data and as median values with interquartile ranges for variables with skewed data distributions. Categorical data were presented as frequencies and percentages. The chi-square test was used to compare between categorical variables while the independent sample *t* test and the ANOVA test were used to compare quantitative variables between groups. *P* values of less than 0.05 were considered statistically significant.

## Results

The demographic data of the participants in the different study groups are presented in Table 1. No significant differences in age, sex, smoking status, and height were observed between the three groups. Phonasthenic manifestations (Table 2) were present in 60.8% of patients with pneumonia, 87.5% of patients with severe pneumonia, and 12% of the control participants with a highly significant difference between the three groups. Table 3 shows highly significant differences in voice quality, register, and loudness between COVID-19 survivors and control participants with no significant difference between the pneumonia and severe pneumonia groups. There was no significant difference in the pitch and glottal attack between the discharged COVID-19 survivors of both groups and the control participants (Table 4). Table 5 shows a highly significant difference in the MPT between the groups; 15.97 ± 3.91 s in the control group, 10.72 ± 4.09 in the pneumonia group, and 8.88 ± 2.68 in the severe pneumonia group.

**Table 1:** Demographic characteristics of the participants in the different study groups

Item	Group 1 “Pneumonia” “*n* = 212”	Group 2 “Severe Pneumonia” “*n* = 152”	Group 3 “Control” “*n* = 100”	*P* value
Sex				
Male Female	103 (48.6%) 109 (51.4%)	75 (49.3%) 77 (50.7%)	48 (48.0%) 52 (52.0%)	*P* = 0.977
Smoking				
Yes No	91 (42.9%) 121 (57.1%)	69 (45.4%) 83 (54.6%)	43 (43.0%) 57 (57.0%)	*P* = 0.640
Age				
Mean ± SD Range	53.71 ± 13.77 (23.0–76.0)	52.4 ± 12.23 (24.0–76.0)	51.15 ± 15.82 (22.0–75.0)	*P* = 0.291
Height				
Mean ± SD Range	168.15 ± 12.42 (148.0–185.0)	166.12 ± 16.27 (150.0–187.0)	167.31 ± 14.17 (150.0–185.0)	*P* = 0.369

Sex & smoking *P* value using Chi-square test, Age & Height *P* value using ANOVA test

**Table 2:** Comparisons of phonasthenic manifestations between the different groups

Item	Group 1 “Pneumonia” “*n* = 212”	Group 2 “Severe Pneumonia” “*n* = 152”	Group 3 “Control” “*n* = 100”	*P* value
Phonasthenic manifestations				
Yes No	129 (60.8%) 83 (39.2%)	133 (87.5%) 19 (12.5%)	12 (12.0%) 88 (88.0%)	*P* < 0.000 *P < 0.000 **P < 0.000 ***P < 0.01

Using Chi-Square test *P; group 1 to group 3, **P; group 2 to group 3 ***P; group 1 to group 2

**Table 3:** Auditory perceptual assessment of the voice: voice quality, register, and loudness in the different groups

Item	Group 1 “Pneumonia” “*n* = 212”	Group 2 “Severe Pneumonia” “*n* = 152”	Group 3 “Control” “*n* = 100”	*P* value
Grade of dysphonia				
0 1 2 3 Total number of patients with dysphonia	179 (84.4%) 26 (12.3%) 4 (1.9%) 3 (1.4%) 33 (15.6%)	125 (82.2%) 19 (12.5%) 6 (3.9%) 2 (1.3%) 27 (17.7%)	100 (100.0%) 0 0 0 0	*P* < 0.002 *P < 0.01 **P < 0.01 ***P = 0.699
Voice quality				
Normal Leaky Rough Strained & Leaky	179 (84.4%) 13 (6.1%) 9 (4.2%) 11 (5.2%)	125 (82.2%) 18 (11.8%) 2 (1.3%) 7 (4.6%)	100 (100%) 0 0 0	*P* < 0.000 *P < 0.001 **P < 0.001 ***P = 0.435
Register				
Modal Modal with vocal fry	139 (65.6%) 73 (34.4%)	97 (63.8%) 55 (36.2%)	91 (91.0%) 9 (9.0%)	*P* < 0.000 *P < 0.000 **P < 0.000 ***P = 0.661
Loudness				
Normal Excessively soft Excessively loud	149 (70.0%) 62 (29.2%) 1 (0.5%)	101 (66.4%) 51 (33.6%) 0	91 (91.0%) 7 (7.0%) 2 (2.0%)	*P* < 0.000 *P < 0.000 **P < 0.000 ***P = 0.488

Using Chi-Square test, *P; group 1 to group 3, ** *p*; group 2 to group 3, ***P; group 1 to group 2

**Table 4:** Comparison of the auditory perceptual assessment of the voice, “pitch & glottal attack,” between the three groups

Item	Group 1 “Pneumonia” “*n* = 212”	Group 2 “Severe Pneumonia” “*n *= 152”	Group 3 “Control” “*n* = 100”	P-value
Pitch				
Normal	212 (100%)	152 (100%)	100 (100%)	–
Glottal attack				
Normal Soft Hard	205 (96.7%) 6 (2.8%) 1 (0.5%)	148 (97.4%) 4 (2.6%) 0	100 (100%) 0 0	*P* = 0.402 *P = 0.185 **P = 0.102 ***P = 0.693

Using Chi-Square test *P; group 1 to group 3, **p; group 2 to group 3 ***P; group 1 to group2

**Table 5:** Comparison of the maximum phonation time between the three groups

Item	Group 1 “Pneumonia” “*n* = 212”	Group 2 “Severe Pneumonia” “*n* = 152”	Group 3 “Control” “*n* = 100”	*P* value
MPT in seconds (mean ± SD) Range	10.72 ± 4.09 (4.55–22.3)	8.88 ± 2.68 (4.74–16.22)	15.97 ± 3.91 (6.00–22.06)	*P* < 0.000 *P < 0.000 **P < 0.000 ***P < 0.000
MPT in Males MPT in Females	11.72 ± 4.53 9.75 ± 3.36	10.42 ± 2.64 7.37 ± 1.67	17.89 ± 4.23 14.05 ± 2.79	*P* < 0.000 *P* < 0.000

Using ANOVA test & independent *T* test *P; group 1 to group 3, **p; group 2 to group 3 ***P; group 1 to group2

## Discussion

The universal outbreak of COVID-19 led to the occurrence of otolaryngological manifestations of this infection, which are mainly related to the loss of smell and taste. However, many other otolaryngological symptoms could be predominant in COVID-19, raising concerns about the assessment of the laryngeal function in recovering patients. There is currently no report on the laryngeal function in discharged COVID-19 survivors. This study aimed to assess the laryngeal function by evaluation of voice quality and MPT of recovered COVID-19 survivors. Phonasthenia is a functional voice disorder in which dysphonia is not heard but felt in the neck and throat by the patients [14]. In this study, 262 (72%) COVID-19 survivors had phonasthenic manifestations in the form of dryness of the throat, easy fatigability of the voice, frequent throat clearance, and throat soreness. We also reported that phonasthenic manifestations had a direct relationship with the severity of illness as they were significantly more frequent in patients with severe pneumonia than in those with pneumonia. The most common predisposing factors to phonasthenia were the misuse and abuse of the voice, smoking, poor physical condition, and generalized fatigue [14, 15]. Generalized fatigue and poor physical condition are common in patients with confirmed COVID-19 [16, 17], that is associated etiologically with phonasthenia [15]. Carfi et al. 2020 found that 53% of COVID-19 survivors experienced fatigue and 43% of them reported dyspnea and shortness of breath [18]. The association between chronic fatigue and voice complaints has been reported in a previous study on young singers [19]. Dysphonia is a change in the vocal quality, pitch, loudness, or vocal exertion that impairs communication. It affects one-third of people at a certain point in their lives and is encountered in less than 20% of common viral infections such as a common cold or flu [20, 21]. In the present study, varying degrees of dysphonia, from mild to severe, with abnormal qualities of the voice (leaky, strained, and rough) were recorded in 15.6% of COVID-19 survivors with pneumonia and 17.7% of COVID-19 patients with severe pneumonia. We found significant differences (*P* < 0.001) between COVID-19 survivors and the control group. In a recent epidemiological study, dysphonia was reported in 26.8% of patients with mild-to-moderate COVID-19 and could be added to the symptomatology of COVID-19 while aphonia was reported in 3.7% of patients [22].

Our findings are in line with those of Jerome et al. who reported that the prevalence of dysphonia in COVID-19 remains lower than that of other common symptoms such as the total loss of smell and taste [20]. Vocal fry register was observed in 34.4% of COVID-19 with pneumonia and in 36.2% of COVID-19 with severe pneumonia. Moreover, excessively soft phonation was observed in 29.2% and 33.6% of patients who had COVID-19 with pneumonia and severe pneumonia, respectively. These findings could be explained by the generalized weakness, fatigue, and shortness of breath that affected their voices and rendered them weak (perceptually). However, there was no significant difference in these auditory perceptual characteristics between the pneumonia and severe pneumonia groups. Phonasthenic manifestations and altered auditory perceptual voice characteristics could be likely related to the laryngeal involvement in the airway inflammatory process and may be caused by cough-related injuries [23]. Coughing slams the vocal folds forcefully together to clear mucus, and cough-related injuries to the vocal folds’ mucosa may affect the vibration of these structures to varying degrees of severity [24]. It is known that respiratory weakness can modify the vocal patterns, influencing the maintainability of vocal production [25] and the efficient use of the breath is essential for healthy phonation [24]. Recent studies have revealed that the lung is the organ that is most affected by COVID-19 [2, 3] which results in an impairment of the phonatory function. Our results show a worsening of the MPT in COVID-19 survivors compared with the control group. The mean MPT was significantly lower in the severe pneumonia group than in the pneumonia group, which suggests higher impairment of the lung volume and phonatory function in severe cases.

In conclusion, our study revealed that in discharged COVID-19 survivors, phonasthenia is a common complaint and its incidence increased with the severity of the illness and that it should be considered a symptom of the disease. Dysphonia may be encountered in patients with moderate-to-severe disease. Physicians have to keep in mind that COVID-19 patients may develop dysphonia throughout the clinical course of the disease as it usually occurs in common viral infection of the upper aerodigestive tract mucosa. The decrease in the MPT among patients was in line with the varying degree of severity of the disease among the patients.

### Limitations

The cross-sectional study design did not allow us ample time for follow-up.

The association between phonasthenic manifestations, the abnormal voice quality, the shortened MPT, and the laryngostroboscopic evaluation was not determined in our study to avoid the risk of contamination associated with the video laryngostroboscopic examination.

### Recommendations

The long-term evaluation of the phonatory function after recovery still requires further investigations that include stroboscopic examinations of the vocal folds. More studies are needed to explore the place of phonasthenia and dysphonia as clinical manifestations of COVID-19, and the etiology of phonatory function impairment in COVID-19 patients requires more investigations.
